# Physical nature enhances creativity, while virtual nature strengthens creative self-concept

**DOI:** 10.3389/fpsyg.2025.1706001

**Published:** 2026-01-12

**Authors:** Maral Jafari Ranjbar, Yongyeon Cho, Sarah Zenti, Stephen B. Gilbert

**Affiliations:** 1 Human Computer Interaction, Iowa State University, Ames, IA, United States; 2 Interior Design, Iowa State University, Ames, IA, United States; 3 Industrial and Manufacturing Systems Engineering, Iowa State University, Ames, IA, United States

**Keywords:** attention restoration theory, creativity, divergent thinking, restorative environments, virtual reality

## Abstract

Enhancing creativity is essential in educational and professional settings. While previous research has demonstrated that physical nature can boost creativity by supporting cognitive restoration, this study explores the effects of both physical nature and virtual nature exposure on creativity. It specifically examines how these environments influence participants’ creative abilities and self-perceived creative identity and compares the relative impact of physical nature vs. virtual nature exposure on creativity. A total of 64 university students in the United States from diverse academic backgrounds were assigned to one of two conditions: exposed to physical nature or virtual nature. Creativity, self-perceived creativity, and restorative perceptions were assessed using the Alternative Uses Task (AUT), Short Scale of Creative Self (SSCS), and Restoration Outcome Scale (ROS). A linear mixed model (LMM) and paired t-tests analyzed changes over time and between groups. Results showed a significant increase in creativity over time in the physical nature group. Both groups reported higher SSCS, with virtual nature participants showing a slightly larger increase. Creativity improvements were positively correlated with restorative outcomes, regardless of nature type. These findings highlight the unique role of physical nature in enhancing divergent thinking while also pointing to the potential of virtual nature to strengthen creative self-concept.

## Introduction and theoretical background

Creativity has become an increasingly vital attribute in personal, professional, and societal development, particularly in developed nations where innovation is closely tied to social and economic progress (Heilman, 2016; Leščinskij et al., 2025). Creativity is defined as the ability to produce original and valuable ideas across diverse domains and has attracted extensive scholarly attention due to its critical role in addressing contemporary challenges (Amabile et al., 1996; Woodman et al., 1993; Edgell and Lee, 2023; Stein, 2014). At both individual and national levels, creativity fosters innovative thinking, problem-solving, and unique approaches to complex issues, contributing to personal achievements and broader societal advancement (Heilman, 2016). Nations that prioritize creativity and innovation experience economic growth, social development, and increased global competitiveness, driven in part by creative individuals who launch businesses, develop technologies, and influence various disciplines (Heilman, 2016; Leščinskij et al., 2025).

The growing emphasis on creativity is further intensified by rapid technological advancements and the transition to a knowledge-driven economy, which challenge traditional models of learning and knowledge acquisition (Valquaresma and Coimbra, 2021; Warner and Myers, 2010). As we enter what Pink (Pink, 2005) refers to as the “conceptual age,”—a period in which success increasingly depends on creativity, empathy, design, and integrative thinking—creativity supports pattern recognition, narrative construction, and the integration of diverse ideas, fostering the development of self-reliant, open-minded, and innovative individuals capable of meeting evolving societal demands (Valquaresma and Coimbra, 2021; Pink, 2005; Stojanova, 2010).

### Frameworks linking creativity and nature

A longstanding question in creativity research is whether creativity can be cultivated or enhanced, and if so, which factors contribute most effectively to its development. While cognitive traits and personality characteristics have often been considered relatively stable, emerging research highlights that creativity can be shaped by external factors such as environment, mood, and attentional resources (Runco, 2004). In parallel, research in environmental psychology has explored how exposure to natural environments may influence cognitive functions, drawing on frameworks such as Attention Restoration Theory (ART). ART posits that nature engages individuals through “soft fascination,” which restores attentional capacity without requiring focused mental effort, thus facilitating cognitive restoration and enhanced creative thinking (Basu et al., 2019; Williams et al., 2018; Kirshbaum, 2021). These restorative effects have been linked to improved cognitive performance, including memory, executive functioning, and creativity (Atchley et al., 2012; Stevenson et al., 2018; Crossan and Salmoni, 2021; Plambech and Konijnendijk Van Den Bosch, 2015). Environments rich in green and blue spaces, such as zoos, aquariums, and botanical gardens, have been associated with enhanced well-being, cognitive functioning, and social interaction, further suggesting that natural settings may positively influence creativity (Maple and Morris, 2018; Trammell and Aguilar, 2021).

Although Attention Restoration Theory (ART) has served as a foundational framework to explain how nature influences cognitive functioning through mechanisms such as “soft fascination,” its theoretical assumptions have faced increasing scrutiny. Some scholars have argued that central constructs within ART, such as soft fascination, remain conceptually vague and under-defined, limiting the framework’s explanatory power and empirical testability (Joye and Dewitte, 2018). Moreover, although meta-analytic reviews have provided some support for ART, particularly in relation to working memory performance, results remain inconsistent across different cognitive measures, suggesting that the restorative effects of nature may vary depending on the specific aspects of attention being assessed (Ohly et al., 2016). This ongoing debate highlights the need for further empirical investigations to clarify the mechanisms underlying nature’s cognitive benefits and to determine whether such restorative effects extend beyond physical nature to virtual natural environments that could be experienced by people when physical nature is not readily available.

In addition to ART, other theoretical frameworks have been proposed to explain nature’s influence on creativity. Stress Reduction Theory (SRT) suggests that exposure to nature reduces physiological stress and arousal, thereby enhancing mood, cognitive flexibility, and creative thinking (Palanica and Fossat, 2022; Ulrich et al., 1991). Additionally, mind-wandering theories propose that natural settings facilitate introspection and spontaneous thought processes, allowing individuals to generate novel associations and solutions through free-flowing cognitive exploration (Williams et al., 2018; Atchley et al., 2012). These complementary perspectives highlight that multiple pathways may underlie the relationship between natural environments and creative cognition.

### Previous research on the impact of virtual nature

Although prior research suggests that natural environments can enhance creativity (Atchley et al., 2012; Plambech and Konijnendijk Van Den Bosch, 2015), most studies have focused either on physical nature or general cognitive and emotional outcomes, rather than directly comparing the cognitive impact of physical and virtual natural environments on creativity specifically. In particular, the relationship between perceived restoration and creativity has received limited attention, despite emerging evidence that immersive virtual environments may evoke similar affective and attentional responses as real nature (Anderson et al., 2017; Smith, 2015).

Recent studies have started to explore the role of virtual nature in promoting creativity. For instance, Palanica et al. compared creative performance following exposure to natural and urban environments presented in three formats: real-world, 2D video, and immersive 3D virtual reality. Their findings showed that nature-based environments, regardless of medium, were associated with greater creativity than urban environments, and that immersive VR scenes could produce creativity outcomes comparable to real-world exposure. However, their study focused on content type (nature vs. urban) rather than format, and did not directly compare the effects of physical and virtual natural environments. Graessler and Taplick (2019) developed a VR-supported creativity tool designed for engineering teams and demonstrated the potential of custom-configured virtual environments for enhancing idea generation, though their study focused on structured product design contexts rather than natural environments.

Other scholars have highlighted the value of immersive Virtual Reality (VR) as a research tool for understanding environmental perception, mood, and behavior (Smith, 2015; Cho and Park, 2023). For example, Anderson et al. (2017) and Newman et al. (2022) found that VR representations of nature could reduce stress and negative affect, suggesting a potential overlap with the restorative mechanisms proposed by Attention Restoration Theory. However, these studies primarily assessed emotional or attentional outcomes and did not measure creativity. Similarly, Cho and Park (2023) demonstrated the educational potential of immersive natural VR simulations, but did not examine their cognitive impact on creativity or restoration. To address these gaps, the present study compares the effects of physical and virtual nature exposure on both objective measures of creativity and self-perceived creative identity, while also assessing whether perceived restoration is associated with creativity outcomes across both settings.

Specifically, this study investigates (Heilman, 2016) differences in creativity levels between participants exposed to physical nature vs. virtual nature following a restorative experience (Leščinskij et al., 2025) the impact of such exposure on self-perceived creative identity, and (Amabile et al., 1996) the relationship between participants’ perceptions of restoration and changes in creativity. By exploring these questions, this study provides new insight into whether immersive virtual environments can meaningfully replicate the cognitive and emotional benefits of physical nature in fostering creativity, especially in contexts where access to real nature is limited.

Based on prior findings and theoretical frameworks such as Attention Restoration Theory and Stress Reduction Theory, as well as evidence that both physical nature and virtual nature can enhance creativity compared to urban environments (Palanica et al., 2019), the following hypotheses were proposed:

- H1: Participants exposed to physical nature will show a greater increase in creative performance (as measured by AUT scores) than participants exposed to virtual nature.
- H2: Participants in the physical nature condition will report a greater increase in self-perceived creative identity (as measured by the Short Scale of Creative Self) compared to those in the virtual condition.
- H3: Perceived restoration (as measured by the Restoration Outcome Scale) will be positively associated with creativity outcomes (AUT and SSCS scores) across both conditions.

## Materials and methods

### Participants

Sixty-four students were recruited from a large US university. Participants were divided into two groups using a quasi-randomized design based on alternating condition assignment based on signup timing: 30 students (approximately 46.9% of the total sample) were assigned to the physical nature group, consisting of 11 men (36.7%) and 19 women (63.3%). The other 34 students (approximately 53.1%) were assigned to the virtual nature group, with 16 men (47.1%) and 18 women (52.9%). True randomization was not feasible due to two practical constraints: (1) campus weather variability, which affected when outdoor sessions could be safely conducted, and (2) VR room scheduling limitations that restricted the flexibility of laboratory-based sessions. As a result, participants were assigned using an alternating sign-up procedure, which ensured balanced group sizes despite these logistical constraints. The sample included both undergraduate and graduate students from diverse academic disciplines. Participants were all adults (18 years or older). Recruitment was conducted via a campus-wide email invitation to participate in a study on creativity and the nature. Interested students completed a brief screening to ensure they met inclusion criteria (e.g., age ≥ 18, comfortable with using VR equipment, and able to walk unaided to an outdoor location). All participants provided IRB-approved informed consent and were compensated with a $10 Starbucks gift card for their participation.

### Measures

#### Creativity (divergent thinking)

Creativity often begins with divergent thinking, a cognitive process that generates diverse ideas spontaneously, exploring broad possibilities and unconventional connections (Runco and Acar, 2012). Divergent thinking ability was assessed with the Alternate Uses Test (AUT), a widely used measure of creativity that evaluates individuals’ capacity to generate multiple, novel uses for common objects (Guilford, 1950). Each participant completed the AUT twice, once before and once after the nature exposure, using two different objects: a *broom* and a *belt* (Alhashim et al., 2020). The object order was counterbalanced across participants to minimize order effects. Participants were instructed to verbally list as many alternative uses for the given object as possible within a 3-min time limit, a duration commonly used in AUT studies to effectively elicit divergent thinking while maintaining practicality (Dippo and Kudrowitz, 2013). All verbal responses were recorded and later transcribed for scoring. Two quantitative indicators of divergent thinking were calculated: fluency, defined as the total number of responses generated, and flexibility, defined as the number of distinct conceptual categories the responses represented (Reiter-Palmon et al., 2019; Torrance, 1966). Originality and elaboration, although part of Guilford’s (Guilford, 1967) classic framework, were not included in this study. Originality scores typically require large normative datasets to benchmark the statistical rarity of responses (Silvia et al., 2008), which was beyond the scope of this sample size. Elaboration, which reflects the amount of detail added to responses, has also been criticized for its weak reliability and inconsistent relationship with creativity outcomes (Silvia et al., 2008). In contrast, fluency and flexibility are widely regarded as the most reliable and theoretically robust indicators of divergent thinking, capturing both the quantity and conceptual breadth of idea generation (Reiter-Palmon et al., 2019; Weiss et al., 2021). For these reasons, this study focused on fluency and flexibility as the primary measures of divergent thinking. Two trained raters independently scored all AUT responses. Raters first met to develop an inductive coding scheme for flexibility by jointly reviewing responses from the first 10 participants. Category definitions were iteratively refined to ensure conceptual clarity, and rare or ambiguous responses were temporarily placed in a miscellaneous category until consensus was reached. After the final category structure was established, both raters applied the coding scheme to all remaining responses, and discrepancies in category assignments were resolved through discussion to ensure scoring consistency. Fluency and flexibility scores were computed separately and then combined into a composite measure of divergent thinking. This decision was supported by the strong correlation between fluency and flexibility at baseline (*r* = 0.886), indicating that both indices reflected a shared underlying construct rather than independent dimensions. Using a composite score also reduced redundancy and minimized the risk of Type I error inflation associated with conducting separate analyses on highly overlapping measures. This scoring approach aligns with established AUT methodologies and provides a stable, theoretically grounded indicator of overall divergent thinking (Weiss et al., 2021; Abdulla Alabbasi et al., 2021).

#### Self-perceived creative identity

The Short Scale of Creative Self (SSCS) was used to measure participants’ self-perceptions of their creativity (Karwowski et al., 2018). The SSCS is a standardized self-report questionnaire that assesses creative self-efficacy and creative personal identity (i.e., one’s confidence in one’s creative abilities and the extent to which one thinks of oneself as a creative person). Participants responded to items on a 5-point Likert scale (rating the extent to which they agree with statements about their creative ability and identity), with response options ranging from *Definitely not* to *Somewhat not*, *Neither yes nor no*, *Somewhat yes*, and *Definitely yes*. Higher SSCS scores indicate a stronger self-perceived creative identity. This instrument has demonstrated good reliability and validity in past research (Wahbeh et al., 2024). In the present study, the SSCS was administered twice (before and after the nature exposure) to examine any changes in creative self-perception as a result of the experimental manipulation.

#### Perceived restoration

Perceived restorative experience was measured with the Restoration Outcome Scale (ROS; Korpela et al., 2008). The 6-item version of the ROS was used, a brief self-report scale that captures the degree to which individuals feel mentally refreshed and restored after an experience. The ROS was completed by participants at the end of the experiment, with items such as “I feel restored and relaxed” rated on a 7-point Likert scale (ranging from *not at all* to *completely*). Higher ROS scores reflect greater perceived restoration. The ROS is a well-established measure in environmental psychology, with proven reliability in assessing the restorative effects of natural environments (Ojala et al., 2019).

### Procedure

**Figure 1:**
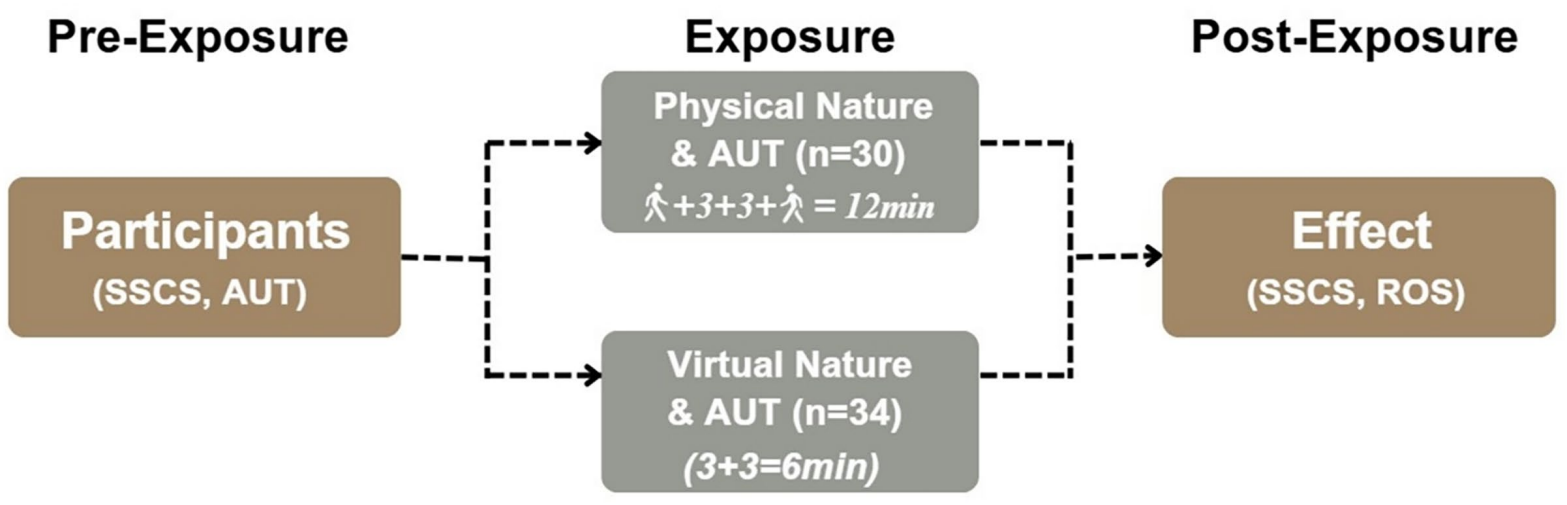
Experimental study design.

This study employed an experimental design consisting of three phases: a Pre-Exposure Phase, a Nature Exposure Phase (physical nature vs. virtual nature), and a Post-Exposure Phase (see Figure 1). Participants were randomly assigned to one of the two experimental conditions: exposure to physical nature or exposure to virtual nature. All sessions were conducted individually, and all experimental appointments were scheduled between approximately 10:00 a.m. and 4:00 p.m. to minimize time-of-day variability in cognitive performance. Both physical and virtual nature sessions were run within this same daily window. At the start of the session (pre-exposure phase), participants completed the baseline measures in a quiet indoor setting. First, they filled out an online survey (via Qualtrics) that included the SSCS to assess their initial self-perceived creative identity. Next, participants completed the first AUT as a baseline creativity measure. They were given one common object word (either “broom” or “belt,” randomly determined) and instructed to verbally list as many alternative uses for that object as possible within 3 mins. The experimenter recorded these responses using a voice recorder for subsequent scoring. This baseline AUT provided a measure of each participant’s divergent thinking performance prior to any nature exposure.

**Figure 2:**
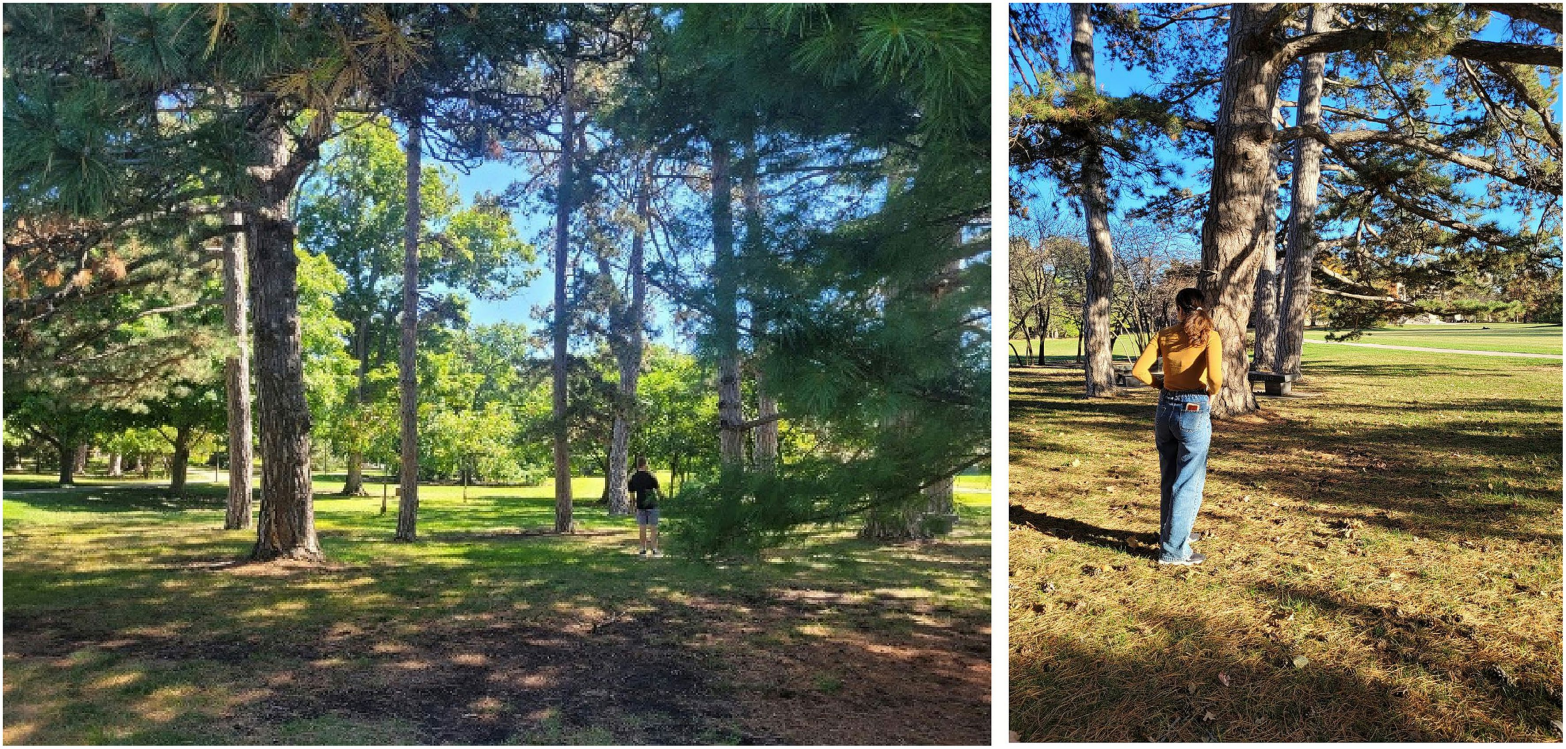
Natural green space located 3 min away from the initial testing room.

**Figure 3:**
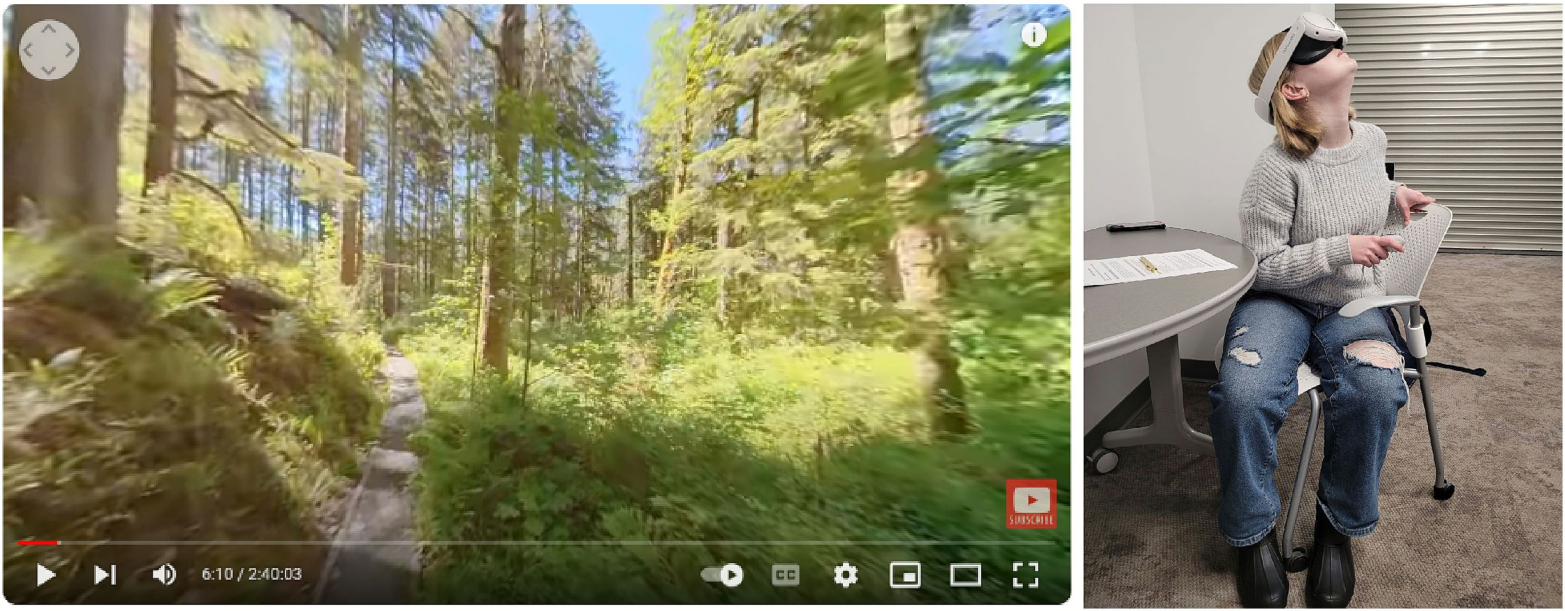
Screenshot of 360° VR video of the Forest Trail Walk used as the video stimulus in the study (adapted with permission from Pro Art Inc. © Pro Art Inc.; source: https://proartwa.com/; original video available at https://youtu.be/q5WUZS-Lyos) and an author-taken photograph of a participant with a VR headset (face blurred).

After the baseline assessments, participants underwent the nature exposure manipulation corresponding to their assigned condition. Participants in the physical nature condition were escorted to a quiet, green outdoor location on campus (approximately a three-minute walk from the initial testing room). Upon arrival, the researcher instructed them: *“You now have three minutes to explore and engage with this environment however you like—feel free to walk around, observe the scenery, or simply relax.”* In this natural setting (see Figure 2), participants spent 3 mins observing the scenery, walking along a short trail, and immersing themselves in the outdoor atmosphere. Outdoor sessions were conducted only during safe weather conditions (no precipitation, no high winds). Across data-collection days, weather was generally mild, ranging from approximately 65–70 °F (18–21 °C), and conditions were typically partly cloudy to sunny. Sessions were postponed when weather conditions fell outside this range to ensure consistency. Participants in the virtual nature condition, by contrast, remained in the lab and were fitted with a VR headset. Before the video began, they were instructed: *“You will remain seated during this experience. You now have three minutes to engage with this virtual environment as if you are in nature—please try to relax and immerse yourself in the experience.”* Then, they viewed a 360° immersive video simulating a nature walk along a forest trail (see Figure 3) for an equivalent duration of about 3 mins. The virtual environment was presented using a Meta Quest Pro headset, which provides a display resolution of 1,920 × 1,800 pixels per eye and an approximate 106° field of view, allowing high-fidelity panoramic imagery. Participants viewed an 8 K UHD 360° hiking video recorded with an Insta360 ONE RS 1-inch 360 Edition camera, and audio was delivered through the headset’s integrated stereo speakers. The VR environment provided a first-person perspective of walking through a lush forest, with audio of nature sounds, to mimic a real nature experience. Participants remained seated for safety but could freely rotate their heads and upper bodies to explore the scene, enabling naturalistic visual scanning. No restrictions were placed on visual exploration, and participants were encouraged to look around naturally during the experience.

Immediately after the three-minute nature exposure, participants completed the second creativity test (post-exposure AUT) in the environment they were currently in. Those in the physical nature condition completed the AUT while still outdoors, and those in the virtual condition completed it while still wearing the VR headset in the lab. For this second AUT, each participant was given the alternate object word that they had not encountered in the pretest (if a participant had “broom” in the first AUT, they were given “belt” in the second, and vice versa). They again had 3 mins to generate as many uses as possible, speaking their ideas aloud to be recorded. This post-exposure AUT aimed to capture any changes in creative performance after experiencing the natural environment (physical or virtual). An important procedural difference between conditions is that participants in the physical nature group were able to walk freely during the exposure period, whereas participants in the virtual nature condition remained seated for safety and hardware reasons. Light physical activity, even at low intensity, has been shown to influence cognitive flexibility and divergent thinking. While movement was not timed explicitly, participants in the physical nature group walked approximately 3 mins from the indoor testing room to the outdoor trail, and most engaged in an additional 1–2 min of light walking between the pre- and post-creativity tasks. Because movement was not recorded or controlled, this difference represents a potential confound in the experimental design and should be considered when interpreting group differences in AUT performance.

Following completion of the second AUT, participants in the physical condition were brought back to the indoor lab, and participants in the virtual condition removed the VR equipment. All participants then proceeded to the post-experiment survey. In this final survey, participants again completed the SSCS (to assess any change in their creative self-concept compared to their baseline) and then filled out the ROS, reflecting on how restorative or refreshing they found the experience they just had. Finally, participants were debriefed, thanked for their time, and given the compensation (a $10 Starbucks gift card) before departing. The total session time for each participant was approximately 20–30 min.

Throughout the procedure, the research team ensured that conditions were as equivalent as possible aside from the nature exposure modality. The timing of tasks was kept consistent, and participants were not informed of the study’s hypotheses or of the other condition to minimize expectation effects. The study was approved by the university’s Institutional Review Board, and all procedures were conducted in accordance with ethical guidelines.

### Data analysis

Three main statistical techniques were employed to analyze the quantitative data collected through the SSCS, the AUT, and the ROS: linear mixed models, t-tests, and linear regression.

#### Linear mixed models

LMMs were used to assess the effects of time (pre- and post-exposure) and environment type (physical nature vs. virtual nature) on creativity scores, with individual participants included as random effects to account for baseline differences in creativity. LMMs are ideal for repeated measures and nested data structures, as they allow for both fixed and random effects, which enhances model precision by accounting for within-subject variability (Snijders and Bosker, 2011).

To clarify the model structure, the AUT analysis included Time (pre = 0, post = 1), Group (physical nature = 0, virtual nature = 1), and their interaction as fixed effects. A random intercept for participants was specified to account for individual differences in baseline AUT performance. Random slopes for Time were not included because each participant contributed only two observations, and random-slope models with very few repeated measurements are known to be unstable, difficult to estimate, and prone to singular fits or overparameterization (Bates et al., 2015). This random-intercept–only structure therefore provided a parsimonious and statistically appropriate model while allowing reliable estimation of the fixed effects. The model can be expressed as:

$\begin{array}{l} {\mathrm{AUT}}_{\mathrm{ij}}=\beta_{0}+\beta_{1}({\text{Time}}_{\mathrm{ij}})+\beta_{2}({\text{Group}}_{i})+\\ \;\beta_{3}({\text{Time}}_{\mathrm{ij}}\times {\text{Group}}_{i})+u_{\mathrm{0i}}+\varepsilon_{\mathrm{ij}}. \end{array}$

Because participants were assigned using a quasi-random alternating procedure, we tested for baseline differences between groups using an independent samples t-test on pre-exposure AUT scores. To further address any potential baseline imbalance, we also ran an additional LMM including baseline AUT as a covariate; however, this adjustment did not change the overall pattern of results. This approach ensures individual differences are controlled, maximizing the model’s ability to detect the main effects of interest. Effect sizes were measured with partial eta-squared ( $\eta_{p}^{2}$ ), with benchmarks of 0.01 (small), 0.06 (medium), and 0.14 (large).

Given that the study used a fixed sample size based on practical constraints, no a priori power analysis was conducted. Instead, a post hoc sensitivity analysis (α = 0.05, two-tailed, 1 – *β* = 0.80) indicated that the available sample size (*N* = 64, two measurements per participant) was sufficient to detect a within-subject Time effect of approximately d ≈ 0.35 in the LMM. For the group-specific paired t-tests, the design was sensitive to pre–post changes of about d ≈ 0.51 in the physical nature group (*n* = 30) and d ≈ 0.48 in the virtual nature group (*n* = 34), suggesting that smaller effects may have gone undetected.

#### Paired samples t-tests

Within each group, paired samples t-tests were conducted to compare pre- and post-experiment AUT and SSCS scores, enabling the study to determine whether significant changes occurred in creativity and self-perception following exposure to nature. This test is suited for comparing two related measurements, as it accounts for dependencies within pairs and effectively tests for mean differences (Cohen, 2013). The required assumptions for t-tests were evaluated (outliers, normality, homogeneity of variance). Effect sizes for t-tests were reported using Cohen’s *d*, with benchmarks of 0.2 (small), 0.5 (medium), and 0.8 (large; Cohen, 2013).

#### Linear regression

Linear regression analysis explored the relationship between restorative outcomes (as measured by the ROS) and changes in creativity, examining whether perceived restoration could predict creativity improvements, regardless of nature exposure type. Linear regression is widely used for examining predictive relationships between continuous variables due to its interpretability and effectiveness in testing hypotheses about predictors and outcomes (Gelman and Hill, 2007). This analysis allowed us to assess the extent to which perceived restoration contributes to creativity, independent of the nature environment.

## Results

### Effect of nature exposure on divergent thinking

**Figure 4:**
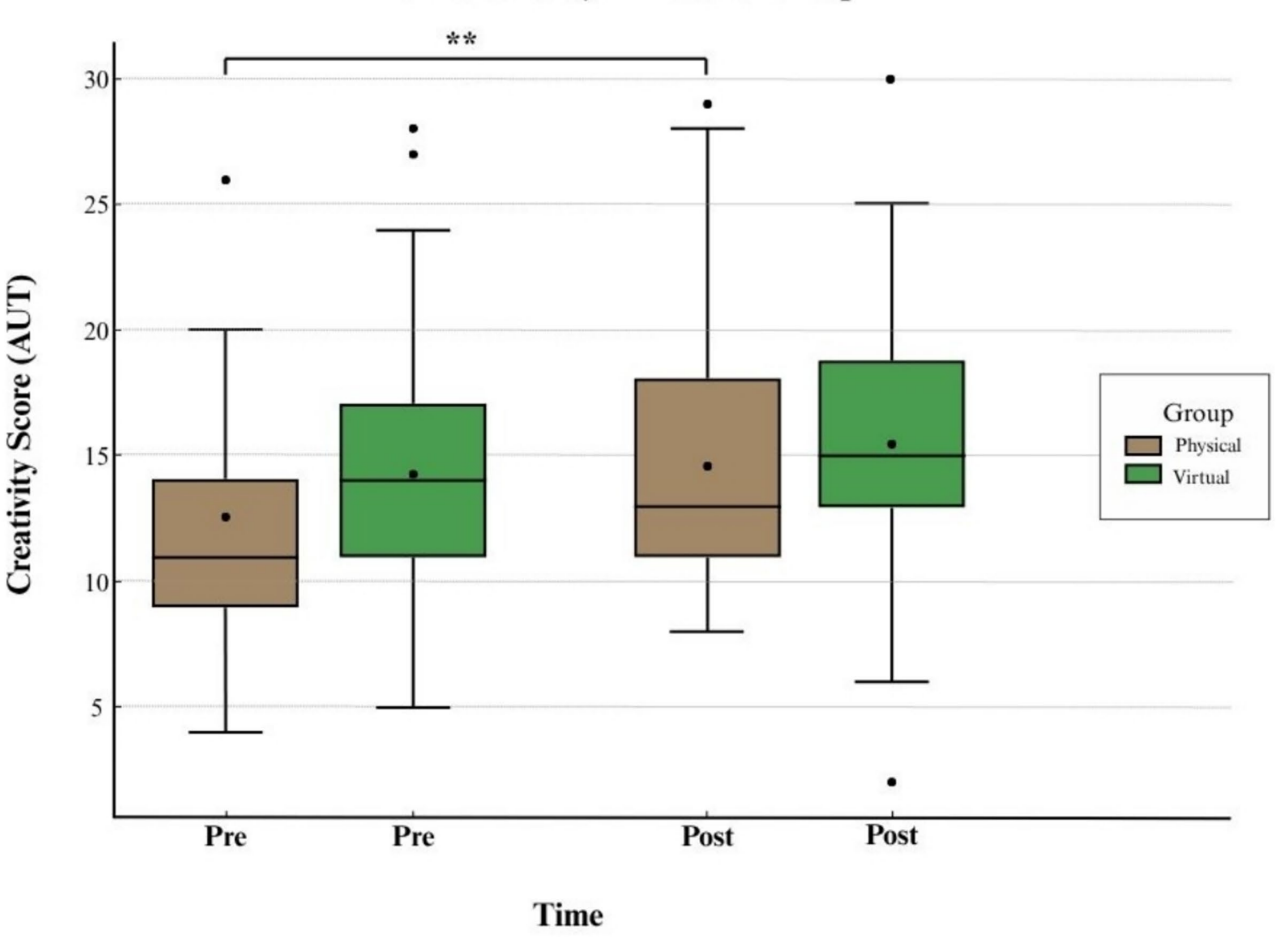
Paired boxplot showing AUT scores by group (physical nature vs. virtual nature) across time (Pre vs. Post).

A linear mixed model (LMM) was used to examine changes in creativity (AUT scores) across time (pre vs. post exposure) and environment type (physical nature vs. virtual nature), with participants included as a random effect to control for baseline variability. Model assumptions were met: residuals were approximately normally distributed, and variance remained constant across fitted values. A paired boxplot (see Figure 4) visualizes changes in creativity across groups and time.

The LMM analysis revealed a significant effect of Time, *F*(1,63) = 7.208, *p* = 0.009, $\eta_{p}^{2}$ = 0.103, indicating an overall increase in creativity following nature exposure. However, the Group effect (physical nature vs. virtual nature) was not significant, *F*(1,62) = 1.112, *p* = 0.296, $\eta_{p}^{2}$ = 0.018, suggesting no differential effect between the two environment types (see Table 1). Random effects analysis confirmed meaningful individual differences in baseline creativity, with *M* = 13.63 and *SD* = 4.37 for pre-exposure AUT scores across both the physical nature and virtual nature groups. To determine whether the two groups differed in divergent thinking at baseline, an independent samples t-test was conducted on pre-exposure AUT scores. The difference between groups was not statistically significant, *t (59)* = 1.24, *p* = 0.220 (Welch’s correction). The physical nature group (*M* = 12.67, *SD* = 5.94) scored slightly lower than the virtual nature group (*M* = 14.44, *SD* = 5.46), but the effect size was small (Cohen’s *d* = 0.31), indicating minimal baseline imbalance. The result was similar when assuming equal variances, *t*(62) = 1.25, *p* = 0.218.

**Table 1:** Effect of time and environment type on creativity improvement in physical and virtual nature groups.

ANOVA summary				
Effect	*df*	*F*	*p*	$\eta_{p}^{2}$
Time	1, 63	7.208	0.009**	0.103
Group (type of nature)	1, 62	1.112	0.296	0.018*

*p < 0.05; **p < 0.01.

Descriptive comparisons of baseline characteristics indicated that the groups were generally similar. Gender distribution was comparable across conditions, with 19 women and 11 men in the physical nature group and 18 women and 16 men in the virtual nature group. No additional baseline variables (e.g., age, prior VR experience, or nature connectedness) were collected.

To further investigate group-specific effects, paired samples t-tests were conducted separately for physical and virtual nature groups. Normality of difference scores was assessed using Q–Q plots and histograms, and no substantial deviations from normality were observed. Visual inspection of boxplots indicated no extreme outliers. Given the paired design, homogeneity of variance between pre- and post-scores was not a requirement, and descriptive statistics confirmed comparable variability across time points, justifying the use of parametric testing.

Physical nature group creativity scores significantly increased from pre- (*M* = 12.67, *SD* = 5.94) to post-exposure (*M* = 14.80, *SD* = 5.69), an increase of 2.13, *t*(29) = 2.910, *p* = 0.003, *d* = 0.53, indicating a moderate effect (see Table 2; Figure 4).

**Table 2:** Paired samples T-test results and descriptive statistics for creativity improvement by group.

Paired samples t-test							
Group	Timepoint	*M*	*SD*	*t*	*df*	*p*	*d*
Physical nature	Pre-Exposure	12.67	5.94	2.910	29	0.003**	0.53
	Post-Exposure	14.80	5.69				
Virtual nature	Pre-Exposure	14.44	5.46	1.274	33	0.106	0.22
	Post-Exposure	15.68	5.34				

*p < 0.05; **p < 0.01.

Virtual nature group scores rose from *M* = 14.44 (*SD* = 5.46) to *M* = 15.68 (*SD* = 5.34), but this difference was not statistically significant, *t*(33) = 1.274, *p* = 0.106, Cohen’s *d* = 0.22 (see Table 2; Figure 4). Physical nature exposure led to a meaningful enhancement in divergent thinking, while virtual nature did not produce a significant improvement.

### Effect of nature exposure on creative self-perception

To assess changes in participants’ creative self-perception, paired samples t-tests were conducted using SSCS scores recorded before and after exposure. Assumptions of normality and absence of outliers were met for both groups.

**Figure 5:**
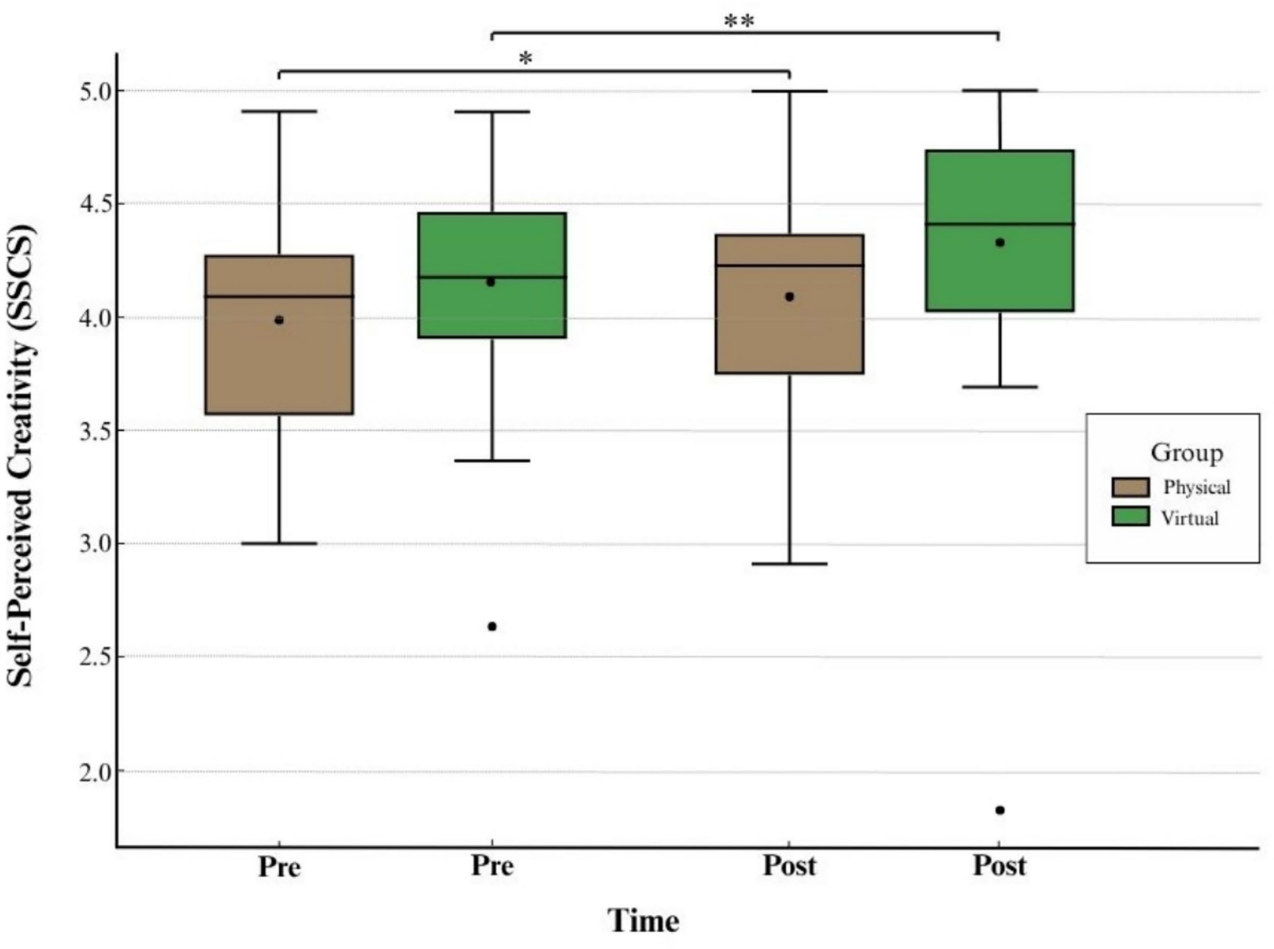
Pre- and post-exposure SSCS scores by group. The differences of the means were statistically significant.

Physical nature group SSCS scores significantly increased post-exposure, *t*(29) = 1.871, *p* = 0.036, *d* = 0.34, a small effect size. Means rose from *M* = 3.99 (*SD* = 0.51) to *M* = 4.09 (*SD* = 0.51), suggesting a modest but meaningful boost in creative self-perception (see Table 3; Figure 5).

**Table 3:** Paired samples T-test results and descriptive statistics for SSCS scores by group.

Paired samples t-test								
Group	Timepoint	*n*	*M*	*SD*	*t*	*df*	*p*	*d*
Physical nature	Pre-SSCS	30	3.991	0.506	1.871	29	0.036*	0.342
	Post-SSCS	30	4.091	0.506				
Virtual nature	Pre-SSCS	34	4.160	0.451	2.809	33	0.004**	0.482
	Post-SSCS	34	4.326	0.595				

*p < 0.05; **p < 0.01.

Virtual nature group participants also reported significantly higher SSCS scores after exposure, *t*(33) = 2.809, *p* = 0.004, *d* = 0.482, a medium effect size. Means increased from *M* = 4.16 (*SD* = 0.45) to *M* = 4.33 (*SD* = 0.60); see Table 3; Figure 5.

Together, these results indicate that both physical and virtual nature environments enhanced participants’ creative self-concept, with slightly larger gains observed in the virtual nature group. To directly examine whether the increase in self-perceived creative identity differed between groups, SSCS change scores (Post–Pre) were compared using Welch’s independent-samples t-test. The analysis showed no significant difference between the physical and virtual nature groups, *t*(61.93) = 0.83, *p* = 0.412, Cohen’s *d* = 0.21. Although the virtual nature condition displayed a slightly larger descriptive increase, the magnitude of improvement did not differ statistically, indicating that both environments produced comparable gains in creative self-concept.

To account for the fact that four paired t-tests were conducted (AUT and SSCS within each group), a Benjamini–Hochberg false-discovery-rate correction (*Q* = 0.05) was applied (Benjamini and Hochberg, 1995). After correction, the pattern of results remained largely consistent with the unadjusted findings. For the AUT analyses, the physical nature group’s improvement remained statistically significant (adjusted *p* = 0.012), whereas the virtual nature group remained non-significant (adjusted *p* = 0.106). For the SSCS analyses, the virtual nature group continued to show a significant increase in creative self-concept (adjusted *p* = 0.008). The physical nature group’s SSCS improvement, while modest, also remained statistically significant under FDR correction (adjusted *p* = 0.048). These corrected results reinforce the conclusion that physical nature reliably improved divergent thinking, and that both physical and virtual nature led to meaningful increases in self-perceived creative identity.

### Relationship between restorative effects and creativity enhancement

To examine whether perceived restoration predicted creativity gains, a linear regression was conducted with creativity improvement (AUT) as the dependent variable and ROS scores and Group (physical nature vs. virtual nature) as predictors. Assumptions for linear regression were met.

**Figure 6:**
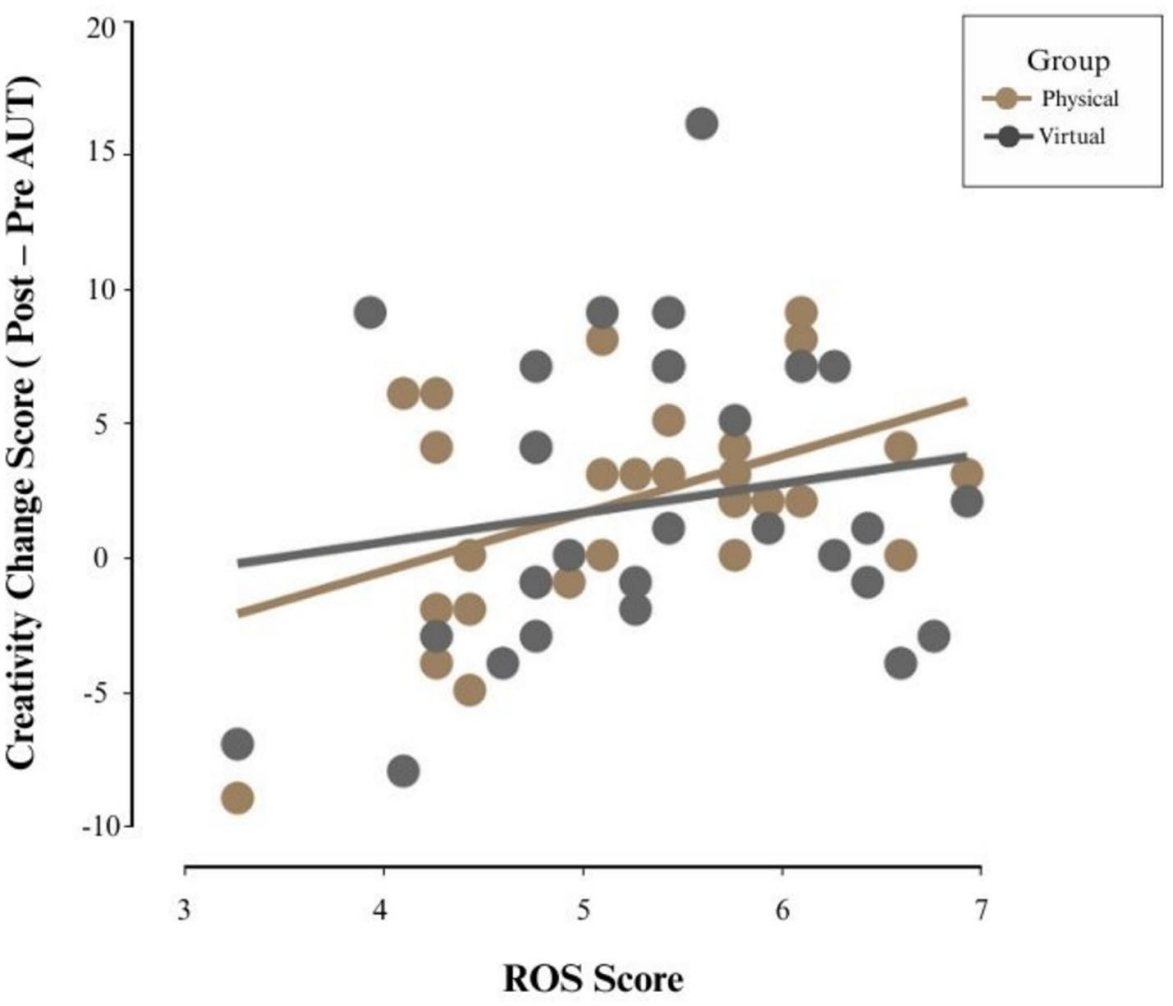
Relationship between restoration ROS scores and creativity improvement (post-pre AUT).

ROS scores significantly predicted creativity change, *B* = 1.600, *t (57)* = 2.315, *p* = 0.024, indicating that participants who felt more restored reported greater creativity improvements (see Table 4; Figure 6). The Group variable was not a significant predictor, *B* = −0.307, *p* = 0.799, suggesting that restoration, rather than environment type, accounted for observed changes.

**Table 4:** Regression results predicting creativity change (AUT) from ROS and group.

Coefficients	*B*	SE	*t*	*p*
ROS	1.600	0.691	2.315	0.024
Groups	−0.307	1.199	−0.256	0.799

These results suggest that restorative experiences can be correlated with creativity enhancement, independent of whether nature exposure occurred in a physical or virtual environment.

## Discussion

This study examined how exposure to physical nature and virtual nature influences creativity (AUT), self-perceived creative identity (SSCS), and perceived restoration (ROS), addressing three hypotheses derived from Attention Restoration Theory (ART), Stress Reduction Theory (SRT), and mind-wandering perspectives.

H1 predicted that participants exposed to physical nature would show greater increases in creative performance than those exposed to virtual nature. Results partially supported this: only the physical nature group demonstrated a statistically significant improvement in AUT scores, while the virtual group showed a non-significant change. This finding is consistent with prior evidence that real-world natural environments rich in sensory input more effectively enhance creativity than virtual simulations (Atchley et al., 2012; Plambech and Konijnendijk Van Den Bosch, 2015). From Attention Restoration Theory (Kaplan and Kaplan, 1989) perspective, physical nature may more fully engage “soft fascination” and multisensory cues, promoting attentional recovery and cognitive flexibility. The lack of improvement in the virtual condition within the current study contrasts with studies that have found creativity gains from VR nature (Palanica et al., 2019) and suggests that, in its current form, VR may lack certain sensory and embodied features necessary for boosting divergent thinking. Specifically, the virtual nature in this study was delivered through a passive 360° video, which restricted participant movement and interaction. Unlike in physical nature, participants could not explore the environment freely, zoom in on specific elements like leaves or textures, or engage in multisensory processing such as feeling the breeze, smelling the foliage, or hearing ambient sounds from multiple directions. This limited sensory fidelity may have constrained the restorative quality of the experience. Furthermore, participants in the VR condition remained seated, whereas those in the physical nature group could walk, an activity known to promote cognitive flexibility and creative ideation (Händel et al., 2024; Mäkelä and Aktaş, 2023).

To address the possibility of a practice effect influencing creativity scores, it is notable that only the physical nature group demonstrated significant gains, despite both groups completing the AUT twice. Previous literature presents mixed evidence regarding practice effects in creativity tasks; while some studies report modest improvement with repetition (Silvia et al., 2009), others show stability in creativity metrics across sessions when no feedback is given (Runco and Acar, 2012). The lack of improvement in the virtual group suggests that practice effects alone are unlikely to explain the creativity gains in the physical nature group.

H2 predicted that physical nature would lead to greater increases in self-perceived creative identity than virtual nature. This was not supported: both groups showed statistically significant increases in SSCS scores. Interestingly, the virtual nature group reported slightly greater gains, suggesting that immersive environments, even when virtual, can positively influence individuals’ perceptions of their own creativity. However, a between-group comparison of SSCS change scores indicated no statistically significant difference between conditions, showing that the magnitude of improvement did not differ reliably across groups. While social desirability or test–retest effects could partially explain this increase, prior research suggests the SSCS is relatively stable across short-term intervals (Karwowski et al., 2018), indicating that these changes likely reflect genuine shifts in self-perception resulting from the nature exposure. The slightly higher gains in self-perceived creative identity in VR—even though the VR participants did not have a significant increase in AUT—may be due to novelty effects, heightened presence, or the self-reflective qualities of immersive environments. It could also be that this incorrect self-perception of increased creativity points a difficulty in creative self-awareness when participants are unfamiliar with reflecting on their creativity levels in detail.

H3 predicted that perceived restoration would be positively associated with creativity gains across both conditions. This was supported: regression analysis showed that higher post-ROS scores significantly predicted greater AUT improvement, regardless of group. This finding supports ART and SRT (Palanica and Fossat, 2022; Ulrich et al., 1991) by highlighting restoration as a cognitive mechanism for creativity enhancement, consistent with recent work underscoring the role of subjective mental refreshment in fostering creative output (Basu et al., 2019).

### Theoretical and practical implications

This study contributes to a growing body of research on how environmental exposure influences creativity, reinforcing ART and related frameworks. The significant improvement in creative performance observed only in the physical nature group suggests that immersive, sensory-rich environments may uniquely engage “soft fascination,” which facilitates attentional recovery and cognitive flexibility (Kaplan and Kaplan, 1989; Fu and Xue, 2023). These findings support the idea that creativity is not solely a mental process but is also shaped by physical surroundings that promote relaxation and spontaneous engagement.

Moreover, the study adds nuance to ART by addressing ongoing critiques regarding its conceptual clarity (Joye and Dewitte, 2018). It suggests that specific environmental features, such as physical movement and direct sensory interaction, may be key to realizing ART’s proposed restorative mechanisms. While both physical and virtual nature were designed to be non-demanding, only the physical group showed significant creativity gains. This distinction may reflect the added benefit of bodily movement and spatial engagement, elements supported by research on embodied cognition and immersive presence (Slater and Sanchez-Vives, 2016; Waterworth et al., 2004).

An additional interpretation is that simply participating in a structured, stress-free study may have served as a brief “mental break,” potentially fostering openness to creative thought. This interpretation is consistent with research suggesting that unstructured time and breaks from goal-directed tasks can enhance creativity (Baird et al., 2012; Sio and Ormerod, 2009; Zedelius and Schooler, 2015). However, if rest alone accounted for the gains, similar improvements would have occurred in the virtual group. The discrepancy implies that environmental features beyond rest, such as physical immersion, may be critical for unlocking cognitive restoration and creative benefits.

While virtual nature did not significantly improve creative performance, it did enhance self-perceived creative identity. This result suggests that VR may still support aspects of creativity, particularly in contexts where access to physical nature is limited. Notably, even in the absence of measurable performance improvements, the immersive qualities of VR appear to shape how individuals perceive their own creative abilities. While this shift in self-concept may not immediately translate into enhanced output, prior research indicates that believing oneself to be creative can increase the likelihood of engaging in creative behaviors and persisting through challenges (Karwowski et al., 2018). This outcome has implications for how virtual environments are designed in education or training contexts, highlighting the importance of presence and perceived engagement in fostering creative confidence.

### Limitations and future research directions

Several limitations should be considered when interpreting this study’s findings. In the virtual condition, participants experienced a fixed-paced, seated VR exposure due to hardware constraints. This lack of control over movement and pacing may have limited immersion and reduced the restorative potential of the virtual environment.

Additionally, the physical nature group engaged in light physical activity (e.g., walking), while the VR group remained stationary. This discrepancy introduces a confounding variable, as physical movement has independently been linked to enhanced creativity through increased cognitive flexibility and relaxation (Händel et al., 2024; Mäkelä and Aktaş, 2023). To further clarify this limitation, it is important to note that only the physical nature group engaged in light walking during the exposure period, making it impossible to attribute creativity improvements exclusively to environmental features of real nature. Movement may have contributed directly to divergent thinking performance. For this reason, future studies should ensure that physical activity is equivalent across conditions or statistically controlled to avoid confounding effects. Future research could conduct a similar experiment in which the virtual nature participants walk around a physical room with a headset, possibly similar to redirected walking studies (Chen et al., 2018).

Beyond the role of physical movement as a confound, it is also possible that embodied cognition processes contributed to the creativity improvements observed in the physical nature condition. Walking outdoors engages multisensory input, rhythmic bodily movement, and naturalistic exploration, all of which have been linked to enhancements in cognitive flexibility and divergent thinking. Such embodied mechanisms may broaden attentional scope and support ideation in ways that are difficult to replicate in a seated VR experience. Thus, the creativity gains observed in the physical condition may reflect the combined influence of environmental stimulation and embodied movement. Future VR-based research could incorporate opportunities for active locomotion or embodied interaction to more closely approximate these processes.

Another important limitation concerns the brevity of the exposure duration. Participants experienced only 3 min of physical or virtual nature, which is substantially shorter than the durations sometimes used in restorative environment research. Although short exposures can yield immediate perceptual or affective benefits, such a brief interval limits the generalizability of the findings to real-world settings in which individuals interact with natural environments for longer or repeated periods. Therefore, the present results should be interpreted as reflecting short-term, transient effects rather than sustained cognitive or creative benefits. In addition, this short exposure duration may have been particularly limiting for the virtual nature condition, as VR-based restoration effects often require longer or more interactive engagement to fully activate attentional and emotional processes. Future studies should examine whether longer or repeated nature exposures produce stronger or more consistent improvements in divergent thinking and creative self-concept. This brief exposure period may have disproportionately constrained the virtual nature condition, as VR-based restoration effects often require longer or more interactive experiences to fully engage attentional and emotional processes.

A further methodological consideration concerns the quasi-random assignment procedure. Because true randomization was not possible due to outdoor weather constraints and VR room availability, participants were assigned based on alternating sign-ups. Although baseline creativity and gender distributions were similar across groups, the absence of complete randomization introduces the possibility of selection bias, such as differences in motivation, availability, or time-of-day preferences. These factors should be considered when interpreting group comparisons.

Another important limitation concerns the absence of baseline measures of participants’ nature connectedness or preference for natural environments. Individuals differ substantially in how strongly they identify with nature or how much they enjoy outdoor settings, and these predispositions may influence both restorative responses and creative outcomes. Without such measures, it is not possible to determine whether pre-existing affinity for nature contributed to the observed effects. Future studies should include assessments of nature connectedness or environmental preference to better account for individual differences in responsiveness to natural and virtual environments.

While virtual nature did not significantly improve creative performance, it did enhance self-perceived creative identity. This suggests promise for VR as a tool to support aspects of creativity, particularly when physical nature access is limited. However, its current limitations, such as restricted movement, fixed pacing, and lower sensory fidelity, may hinder its restorative potential.

Finally, the study’s modest sample size limits statistical power, particularly for detecting small between-group differences. The *post-hoc* sensitivity analysis indicated that the sample was adequate for detecting medium within-subject effects but may have been underpowered to identify more subtle relationships, which should temper the interpretation of non-significant findings. Because multiple statistical tests were conducted, the risk of Type I error was also considered. A Benjamini–Hochberg false-discovery-rate correction was applied to the paired t-tests in the Results section, and the overall pattern of findings remained consistent with the unadjusted analyses. Nonetheless, all results should be interpreted with appropriate caution, and effect sizes are reported throughout to support transparent evaluation of the study’s conclusions. Future studies should develop adaptive VR environments that allow for user-controlled pacing and untethered movement, potentially improving immersion and creative outcomes. Comparative designs that vary physical movement (e.g., seated vs. walking conditions across environments) would help isolate the effects of locomotion vs. environmental exposure. Researchers could also investigate which specific sensory cues from physical nature—such as tactile feedback, multidirectional ambient sounds, and opportunities to visually explore details like leaves or textures—are most critical for enhancing creativity, so that these can be better replicated in VR. Additionally, future work could test whether allowing active exploration in VR, rather than passive video viewing, increases attentional restoration and divergent thinking. Finally, incorporating multisensory and interactive VR elements, such as scent (Baktash et al., 2024), sound, or simulated walking, could enhance presence and better replicate the restorative and creative benefits of real-world nature.

## Conclusion

This study provides preliminary evidence that a brief three-minute physical nature exposure may enhance divergent thinking, supporting Attention Restoration Theory’s proposition that immersive, multisensory environments promote attentional recovery and cognitive flexibility. While virtual nature exposure did not yield significant creativity gains, it did improve self-perceived creative identity, indicating that immersive experiences, even when simulated, may positively influence how individuals view their creative potential. However, a statistical between-group comparison of SSCS change scores showed no significant difference between conditions, indicating that virtual nature did not improve creative self-concept more than physical nature. Both environments produced comparable increases in self-perceived creative identity, suggesting that they supported positive shifts in how participants viewed their creative potential rather than one being superior to the other. The relationship between perceived restoration and creativity across both conditions reinforces the restorative pathway as a key mechanism underlying creative enhancement. These findings suggest that while VR may hold promise as a scalable creativity-support tool, its design must better emulate the embodied, interactive, and sensory-rich qualities of physical environments. Future research should refine VR experiences to address current limitations in movement, pacing, and presence, and further explore how environmental context shapes both creative performance and perception.

## Data Availability

The datasets presented in this study can be found in online repositories. The names of the repository/repositories and accession number(s) can be found at: DOI 10.17605/OSF.IO/NAJ98.
